# A National Surveillance Survey on Noncommunicable Disease Risk Factors: Suriname Health Study Protocol

**DOI:** 10.2196/resprot.4205

**Published:** 2015-06-17

**Authors:** Ingrid SK Krishnadath, Christel CF Smits, Vincent WV Jaddoe, Albert Hofman, Jerry R Toelsie

**Affiliations:** ^1^ Department of Public Health Faculty of Medical Sciences Anton de Kom University of Suriname Paramaribo Suriname; ^2^ Department of Epidemiology Erasmus University Medical Center Rotterdam Netherlands; ^3^ Department of Physiology Faculty of Medical Sciences Anton de Kom University of Suriname Paramaribo Suriname

**Keywords:** ethnicity, multistage cluster sample, noncommunicable disease risk factors, STEPwise approach to surveillance, Suriname

## Abstract

**Background:**

Noncommunicable diseases (NCDs) are the leading cause of death in low- and middle-income countries. Therefore, the surveillance of risk factors has become an issue of major importance for planning and implementation of preventive measures. Unfortunately, in these countries data on NCDs and their risk factors are limited. This also prevails in Suriname, a middle-income country of the Caribbean, with a multiethnic/multicultural population living in diverse residential areas. For these reasons, “The Suriname Health Study” was designed.

**Objective:**

The main objective of this study is to estimate the prevalence of NCD risk factors, including metabolic syndrome, hypertension, and diabetes in Suriname. Differences between specific age groups, sexes, ethnic groups, and geographical areas will be emphasized. In addition, risk groups will be identified and targeted actions will be designed and evaluated.

**Methods:**

In this study, several methodologies were combined. A stratified multistage cluster sample was used to select the participants of 6 ethnic groups (Hindustani, Creole, Javanese, Maroon, Chinese, Amerindians, and mixed) divided into 5 age groups (between 15 and 65 years) who live in urban/rural areas or the hinterland. A standardized World Health Organization STEPwise approach to surveillance questionnaire was adapted and used to obtain information about demographic characteristics, lifestyle, and risk factors. Physical examinations were performed to measure blood pressure, height, weight, and waist circumference. Biochemical analysis of collected blood samples evaluated the levels of glucose, high-density-lipoprotein cholesterol, total cholesterol, and triglycerides. Statistical analysis will be used to identify the burden of modifiable and unmodifiable risk factors in the aforementioned subgroups. Subsequently, tailor-made interventions will be prepared and their effects will be evaluated.

**Results:**

The data as collected allow for national inference and valid analysis of the age, sex, and ethnicity subgroups in the Surinamese population. A publication of the basic survey results is anticipated in mid-2015. Secondary results on the effect of targeted lifestyle interventions are anticipated in late 2017.

**Conclusions:**

Using the data collected in this study, the national prevalence of NCD risk factors will be approximated and described in a diverse population. This study is an entry point for formulating the structure of NCD prevention and surveillance.

## Introduction

### Background

 A noncommunicable disease (NCD) is a medical condition or disease that has a prolonged course, and is neither infectious nor transmissible among people. Worldwide, NCDs, like cardiovascular disease, cancer, chronic respiratory disease, and diabetes are responsible for a large number of deaths. In 2013, the NCD Alliance reported that NCDs account for 60% (35 million) of global deaths and the largest burden—80% (28 million)—occurs in low- and middle-income countries [1]. NCDs and their risk factors worsen poverty, while poverty contributes to rising rates of NCDs, posing a threat to sustainable development [2-4]. It is expected that by 2030 low-income countries will have 8 times more deaths due to NCDs than high-income countries [5]. Compared with industrialized countries, NCD-related deaths occur more frequently and at earlier stages in low- and middle-income countries. In developed countries, 13% of the NCD-related deaths occur before the age of 60. This number is higher (29%) in developing countries [1].

Preventable risk factors are at the root of most NCDs. Worldwide, the leading risk factors for mortality are raised blood pressure (13%), followed by tobacco use (9%), raised blood glucose (6%), physical inactivity (6%), and overweight, including obesity (5%) [1]. Studies in developing countries focused on NCDs and their risk factors, which are important for the identification of subgroups that are at increased risk [3], development of preventive strategies, and eventually to reduce the expected burden of NCDs in the near future. The World Health Organization (WHO) has developed a simple, standardized method for collecting, analyzing, and disseminating data on its member countries: the WHO STEPwise approach to surveillance (STEPS) [6]. More than 90 countries have published their STEPS results in country reports, data books, fact sheets, journal articles, presentations, or posters [7].

### Suriname

The Republic of Suriname is located in the Northeast of South America and has a population of 541638 inhabitants, which is mainly concentrated in the coastal areas [8]. The overall population density is 3.3/km^2^ and ranges from 1.324/km^2^ in the Paramaribo District to 0.3/km^2^ in the Sipaliwini District [8]. The main economic activities in Suriname are gold and bauxite mining, crude oil drilling, agriculture, fishery, forestry, ecotourism, commerce, services, and industry [9]. The gross national income per capita is approximately US $8800/annum [10], which places Suriname among the upper-middle-income countries in the World Bank’s list of economies [11].

The historical development of the country has resulted in a unique social structure, composed of a variety of cultures, religions, ethnicities, and economic units. As a former colony, Suriname has a history of changing ownership that in the end, from 1667 to 1975, remained Dutch. Throughout this period, the Dutch imported slaves from Africa and indentured laborers from China, India, and Indonesia. These groups, together with the settlers and the original inhabitants, the Amerindians, are the ancestors of the present-day population of Suriname [12,13]. At present, the descendants from India are the Hindustani and those from Indonesia are the Javanese. The descendants from Africa are culturally divided into Creoles, descendants of plantation slaves, and Maroons, descendants of refugees who escaped slavery and formed independent settlements. Nowadays, migration is a global phenomenon on the rise [14]. The present population distribution in Suriname is as follows: Hindustani (27.4%), Maroons (21.7%), Javanese (15.7%), mixed (13.4%), and other ethnicities (8.2%) [8].

Suriname’s mortality data underline the burden of NCDs as observed in many middle- and low-income countries. For decades, NCD-related mortality has been reported as the main cause of death in Suriname [15-17]. Currently, the only population data available are collected by the Bureau of Public Health [15-17]. The lack of data on risk factors and morbidity is a major hurdle for the development of preventive strategies.

According to the WHO, surveillance is essential for evidence-based public health decision making and the monitoring of the success of public health interventions [18]. For NCDs, this includes the ongoing systematic collection and analysis of data to provide appropriate information about disease burden, groups at risk, estimates of risk factors, and determinants, coupled with the ability to track health outcomes and risk factor trends over time. Surveillance is critical to provide the information needed for formulation of policies and the development and management of prevention and control programs. It is also basic to measure progress made in implemented policies and programs by monitoring and evaluation [19].

Several studies have reported ethnic differences in cardiovascular disorders and diabetes [20-25]. In Suriname, a 2003 study showed that the highest prevalence of hypertension in adults has been observed in Creoles [26]. However, in another study, high blood pressure was reported to be more frequent in the adolescent Hindustani population compared with other ethnicities [27]. The 2003 study also showed that the prevalence of the combination of hypertension, diabetes, and hypercholesterolemia in adults was higher for Hindustani [26]. Furthermore, a study on 637 patients with diabetes in 12 primary health care centers reported an earlier onset of diabetes in Hindustani (44 years) compared with Creoles (53 years) [28], which indicates a difference among ethnicities.

Data collected in 2001 from 1654 persons (18-55 years) in 3 coastal districts indicated a prevalence rate of 10% for diabetes mellitus, 33% for hypertension, and 5% for both [29]. The survey also provided insight regarding lifestyle and behavioral factors with regard to NCDs: 70% were physically inactive, 30% smoked; 20% were obese, and 15% had high total cholesterol levels.

Adverse lifestyle habits have also been assessed in younger populations. The Global School-Based Student Health Survey 2009 among students aged 13-15 years showed that 73% of the respondents had less than 1 hour of physical activity/day and 81% had a high calorie intake [30]. The 2009 Global Youth Tobacco Survey reported that among students aged 13-15 years, 19.2% were current users of tobacco products. In addition, many students were exposed to “second-hand smoke”: 46.7% lived in homes where others smoked and 53.3% were exposed to smoke outside of their homes [31]. Studies on harmful use of alcohol indicated that among students aged 13-15 years, 73.8% had their first drink before 14 years and 32.6% consumed alcohol on one or more occasions in the past month. Alcohol use was the highest in the 26-34 age group (36.8%), followed by the 35-64 age group (33.9%) [22,30].

The principal objective of this study is to provide baseline data for the monitoring of NCD risk factors. The study will determine the prevalence of NCD risk factors in the age category of 15 and 65 years of 6 ethnic groups living in different geographical areas. Main inquiries such as the national prevalence of metabolic syndrome, diabetes, and hypertension; the national prevalence of NCD risk factors such as tobacco and alcohol use; fruit, vegetable, oil, and fat consumption; physical activity and mental distress; overweight; obesity; and raised blood pressure; the national prevalence of deviating values for biochemical markers such as levels of blood glucose and blood lipids; the presence of age, ethnic, and geographical differences in NCD outcomes and risk factors will be answered. Evidence of ethnic differences in disease burden emphasizes the importance of data segregation to identify risk groups. The study will provide national baseline data on morbidity, which will enable monitoring and evaluation of public health intervention programs. Forthcoming results of this study will also be of interest in nations where similar ethnic groups are present [14].

## Methods

### Overall Design

A multistage cluster, household population cross-sectional design was used in this study. The research proposal for this study was approved by the Ethics Committee of the Ministry of Health of Suriname. Data were collected during the period from March 1 to September 31, 2013. Each study participant was first informed about the details of the study, and then asked to sign a consent form. Besides the aim and survey procedure, the respondent was also explained how the information gathered would be used. The informed consent form consisted of two parts: a form for Steps 1 and 2, concerning the questionnaire and physical measurements and a form for Step 3, concerning the biochemical measurements. The respondent was also explained that he or she could refuse to participate at any period of the study.

All the measures registered in Steps 2 and 3 were revised by medical doctors. All the respondents received the results of their physical and biochemical examinations in writing. The medical staff of the research team provided advice for respondents with an adverse outcome and referred them to the general practitioner.

For the estimated outcome prevalence of 0.5 for the baseline indicators, a sample of 5 10-year age groups between 15 and 65 years from each sex was chosen. For a 95% CI with a margin of error of 0.05, a basic sample requirement of 384.16 was required. With a design effect of 1.5 for the multistage cluster design and 10 sex/age groups, 5762 samples were required.

### Sampling Procedure

**Figure 1:**
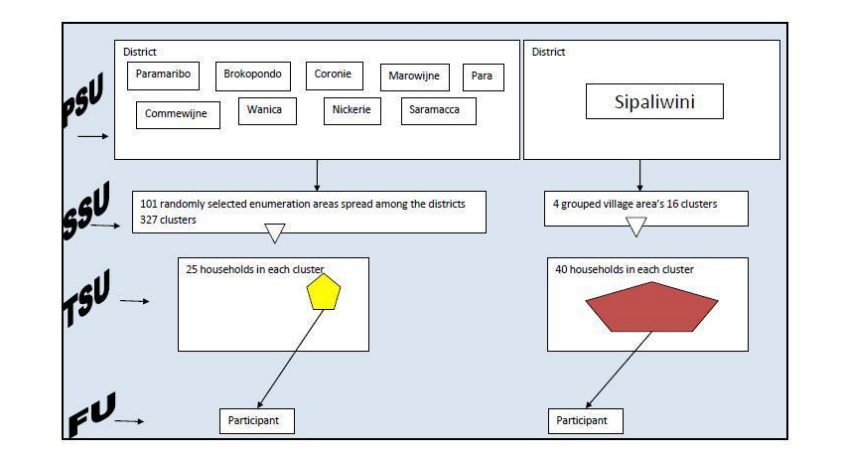
Multistage cluster sample of the Suriname Health Study. FU=Final Unit; PSU=Primary sampling Unit; SSU=Secondary sampling Unit; TSU=Tertiary sampling Unit.

Each of the 10 districts of Suriname served as a primary sampling unit (PSU), and for every PSU a sampling frame was created. In 9 districts, the enumeration areas (EAs) of the Census 2012 were listed [8]. The tenth district, Sipaliwini, included specific village areas (VAs; Figure 1). From the PSUs (n=10), 101 EAs and 4 VAs were selected at random. From these areas, 343 clusters were randomly selected. Within the EAs, each cluster contained 25 households and in the VAs every cluster contained 40 households. The clusters in the VAs were larger because of the high costs associated with reaching the population in this area. In each selected household, the final unit (the respondent) was selected using the Kish method [32] (Figure 1).

The sample size also needed to be adequate to separately analyze each of the 6 ethnic groups, which are as follows: the Creole group, the Hindustani or East Indian group, the Javanese group, the Maroon group, the Amerindian group, and the mixed group. Considering the population numbers, it was estimated that the sample would result in a small number of the Amerindians. Therefore, an oversample of 10 extra clusters from areas with a high density of Amerindians was drawn.

**Figure 2:**
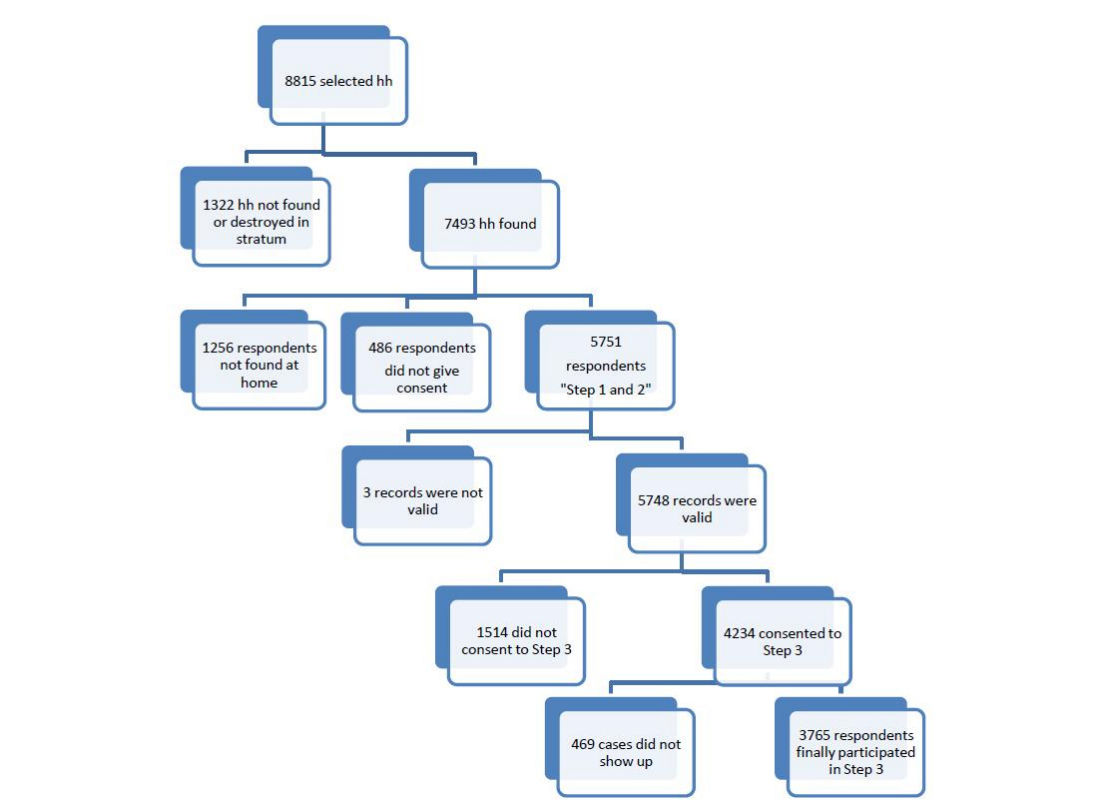
Sample selection flowchart. hh=household.

Within the 343 clusters, 8815 households were randomly selected, and 7493 were invited to participate in the study. The overall response rate was 76.75% (5751 participants). Of these respondents, only 3765 gave a blood sample for analysis. The respondents who have lived in Suriname for less than 1 year and those with health issues that rendered them incapable of participating in the study were excluded (Figure 2).

### Data Collection

#### Overview

Data were collected using the WHO STEPS to chronic disease risk factor surveillance. This method retrieves information on risk factors within a population, and includes different research tools. These tools are used in 3 levels of data collection, described as Steps 1-3 [33,34]. The following 3 steps were conducted in this survey:

#### STEP 1 (Questionnaire)

Information on demographics; smoking; alcohol consumption; dietary habits such as salt, fruit, and vegetable intake; physical activity; the cost of health care and loss of productivity; history of hypertension, diabetes, screening for cervical and breast cancer, injuries, and violence; mental health; and the use of health care was collected using a questionnaire. Apart from income, the other variables corresponding to these questions were composed using 5436/5748 to 5748/5748 of valid data. Only 4052/5748 of the data on income were valid (see Multimedia Appendix 1).

####  STEP 2 (Physiological Measurements)

Body weight and height, waist circumference, and blood pressure, were measured using specific tests and devices. The amount of valid records for these measures ranged from 5423/5748 to 5688/5748 (see Multimedia Appendix 2).

#### STEP 3 (Laboratory Analysis)

Levels of blood glucose, cholesterol, and triglycerides were measured by full-blood analysis. For the analysis of blood-glucose data, 3323/3765 of laboratory data provided valid data, whereas for blood-lipids on average only 3017-3030 of the total valid 3765 records could be used (see Multimedia Appendix 2).

Within the database, extreme outliers and missing data were considered invalid and were subsequently not used in the analysis. The staff participating in this study were trained extensively according to the WHO STEPS manual [35].

### Residential Area and Ethnicity

Based on criteria of the General Bureau of Statistics used in the Suriname Multiple Indicator Cluster Survey on health indicators for children, residential addresses were divided into urban areas, rural coastal areas, and the rural interior according to the residential areas (Table 1) [36].

**Table 1:** Residential areas of Suriname. Source: Suriname Ministry of Social Affairs and Housing, General Bureau of Statistics Suriname. Suriname Multiple Indicator Cluster Survey 2010, Final Report. Paramaribo, Suriname: General Bureau of Statistics Suriname; 2013 [36].

Strata	Districts and resorts
Urban	Paramaribo, Wanica, Nickerie (resort: Nw Nickerie), and Commewijne (resorts: Meerzorg and Tamanredjo)
Rural in the coastal area	The remainder of Nickerie, the remainder of Commewijne, Coronie, Saramacca, Para, and Marowijne
Rural in the interior	Brokopondo and Sipaliwini

Ethnicity has a racial and a cultural component. Self-reported ethnicity of the individual was shown to be deficient when evaluating health components [37-40]. Thus, to determine the ethnicity of a participant both self-reported ethnicity and deduced ethnicity would be used. For deduced ethnicity, that of the grandparents will be considered. A person was categorized into a certain group if at least three of the four grandparents will be considered of ethnicity of that specific group. All others will be categorized as “mixed ethnicity.” Self-reported ethnicity will be used only for comparison with other data collected using the same method (eg, to adjust for ethnicity with regard to the census data [8]).

### Physical Measurements and Equipment

Respondents were measured and weighted as described in the WHO STEPS manual Part 3 [35]. Blood pressure was measured 3 times with the Omron HEM-780 blood pressure monitor. Height was measured with the Seca 213 stand-alone stadiometer, waist with the Seca 201 measuring tape, and weight with the Tanita HS302 solar scale.

### Biological Samples

We signed a contract with a commercial laboratory in Paramaribo (ISO 9001:2008 certified) for performing all the biochemical analyses. Blood samples were collected from the respondents after they fasted for 12 hours overnight. The samples were drawn at home or at a nearby place to increase response. For respondents who failed the 12-hour fasting blood test, the number of hours fasted was registered and their blood was drawn. All blood samples were collected in sodium fluoride (NaF) tubes (2 mL) and lithium heparin (LiHep) tubes (4 mL) for the analysis of glucose and cholesterol levels, respectively. Each sample was labeled with a barcode, which corresponds to the name of the respondent. In the laboratory, the LiHep and NaF tubes were centrifuged at 4100 rpm for 8 minutes at 20°C. The biological samples were analyzed using a CX9 fully automated analyzer (Beckman Coulter, Inc, Atlanta, GA).

The drawn blood was stored in a cooler with ice packs (temperature between 6 and 20°C) and transported to the laboratory within 4 hours. In remote areas, the blood was centrifuged at 4100 rpm for 8 minutes and stored between 3 and 8°C while waiting for transportation to the central laboratory. Once the samples arrived at the laboratory, they were processed and analyzed. Before performing the analysis, 1 mL of the LiHep plasma sample was pipetted into cryo vials to establish a bank of sera for this study. These aliquots were then stored at −80°C.

### Interventions and Follow-Up

After data analysis, risk groups will be identified and targeted by custom-fitted lifestyle interventions, which will be implemented within 6 months after the basic study results are made available. The stored aliquots (from the serum bank) will be analyzed as baseline data to measure the effectiveness of interventions. The effects of all interventions will be assessed and disseminated after 2 years of implementation. For surveillance, the basic set up of this study will be repeated after 5 years.

### Data Management

After data collection, all questionnaires were verified for completeness and consistency of responses. In addition, we evaluated the reliability of the interviewer by partially reinterviewing 238/4757 of the respondents at random in the coastal area. EpiData was used for data entry, which started in April 2014. Frequent quality checks were performed to detect and correct errors in data entry. The laboratory results of the biochemical analysis were added to the file of the respondent. The data entry and validation of these results were done separately. Double data entry resulted in two databases, which were crosschecked using Epi Info’s data compare tool. Finally, 2 identical databases, K1 and K2, were created. Once data entry was complete, it was prepared for cleaning and analysis. The unique identification code for the variables included a code for location, which enabled us to trace each respondent. This code was used to divide the sample into rural and urban localities as presented in Table 2.

**Table 2:** Data by urban or rural area for Steps 1-3.

Areas	Steps 1 and 2 n (%)	Step 3 n (%)
Urban areas	2797 (48.7)	1750 (46.6)
Rural coastal areas	1959 (34.1)	1321 (35.0)
Rural interior areas	992 (17.3)	694 (18.4)
Total	5748 (100.0)	3765 (100.0)

The variables age and sex were checked and only those records containing both variables were considered valid for additional analysis because both variables are needed to analyze the survey data by age-sex groups. Records with either one of these variables missing were considered invalid. Table 3 provides an overview of respondent’s sex by age group for the sample in each step of the survey, and Table 4 presents this overview for ethnicity. Outliers in the data were revised and if incorrect, they are registered as missing. Results deemed unusual, but nevertheless correct were left in the database.

**Table 3:** Valid data presented by sex and age group for Steps 1-3.

	Steps 1 and 2			Step 3		
Age group	Men	Women	Total	Men	Women	Total
15-24	421	670	1091	240	433	673
25-34	426	857	1283	213	581	794
35-44	493	794	1287	286	567	853
45-54	491	728	1219	318	523	841
55-64	324	544	868	203	401	604
Total	2155	3593	5748	1260	2505	3765

**Table 4:** Frequency of data by ethnic groups.

Ethnic group	Steps 1 and 2 n (%)	Step 3 n (%)
Creole	693 (12.1)	445 (11.9)
Hindustani	1342 (23.5)	916 (24.5)
Javanese	935 (16.4)	600 (16.0)
Maroon	1395 (24.5)	943 (25.2)
Amerindian	435 (7.6)	300 (8.0)
Mixed ethnicity	833 (14.6)	499 (13.3)
Other ethnic groups	74 (1.3)	39 (1.0)
Total	5707 (100.0)	3742 (100.0)

### Weighting of Subgroups

Collected data will be subjected to a weighting procedure so that inferences can be made to the whole population. The weights used for analysis were calculated to adjust for probability of selection, nonresponse, and differences between the sample population and target population (see Multimedia Appendix 3). The nonresponse weight was separately calculated for each district by age group (see Multimedia Appendices 4 and 5). The data will be weighted based on selection and nonresponse. These were applied for individual districts and normalized.

A quick analysis of the data showed that the variability of the response rate for Steps 1 and 2 was quite similar, but differed significantly from the variability of the response rate for Step 3. The difference in variability meant that the weights to be applied for Steps 1 and 2 are different from those to be applied for Step 3. The population data used to calculate weights were deduced from the Census 2012 report [8] (see Multimedia Appendices 6 and 7). The weights of the population size were calculated by age and sex/10-year age group.

Finally, the data will be adjusted for the distribution of the ethnic groups indicated in the research questions. The overall weight of the data is the multiplication of the sample design weight, the response weight, and the adjustment weight (see Multimedia Appendix 8).

### Results

Data collection resulted in 5748 valid data for analysis. These results are anticipated in mid-2015. A report with general tables will be presented to the Suriname Ministry of Health. Results on the effectiveness of targeted lifestyle interventions are anticipated in late 2017.

## Discussion

### Preliminary Findings

The high mortality as a consequence of NCDs necessitates the need for data with regard to their risk factors. In response to this growing need, the WHO STEPS [33] was developed. The use of the same standardized questions and protocols by countries provides information to monitor trends within the country and make comparisons possible between countries. This study was designed to obtain data in order to represent the ethnic and geographic diversities of the Surinamese population by sex in 5 different age groups. For each of these groups, the basic sample requirement of 384.16 is needed for results within a 95% CI. For an estimated design effect of 1.5, a total of 5762 respondents were needed, and finally, 5748 valid questionnaires (99.76%) were entered into the database. Therefore, it can be concluded that this study, as designed, includes an adequate dataset. The analysis of these data, in general and in subgroups, will provide high-precision outcome measures for Steps 1 and 2. However, recall bias and, to a lesser extent, interviewer bias should be considered.

By contrast, the sample size for Step 3 is limited. For the sample size of 3742 (95% CI), no design effect can be considered. This smaller sample size is of consequence for the analysis of subgroups such as age and ethnicity. The male subgroups in Step 3 are under the size of 385 (range 203-313). In addition, the Amerindian ethnic group includes only 300 respondents in Step 3. If the CI is changed from 95% to 90%, then the basic sample requirement will become 270.67. For an estimated design effect of 1.38, however, a total of 3742 would be needed. Therefore, although the size of the sample in Step 3 will result in less-precision outcome values, they are still of considerable value for this study.

The use of normalized weights for inference of these data allows for the presentation of reliable record for the Surinamese population. Further, the results allow for comparisons between ethnicities and geographical areas of various countries. Ethnic differences have been reported in several STEPS surveys [7]. However, in this case, Suriname will be the first country that contributes results for 6 ethnic groups living within one environment. Because of the composition of the Surinamese population, the results can also be used as estimations of prevalence of immigrating ethnicities elsewhere.

### Conclusions

In summary, this study reaches its purpose and presents valid and precise data of the Surinamese population by sex, age group, urban and rural localities, and ethnicity. The study design as realized allows for valid approximations of the prevalence of risk factors for NCDs. Risk groups will be identified, and targeted interventions will be implemented and evaluated. For NCD surveillance, the repetition of such a cross-sectional population survey every 5 years is recommended [6]. This will allow the following of trends; in addition, it is also necessary to evaluate, implement, and adapt health interventions.

## Appendix

Multimedia Appendix 1 Quality of data records for Step 1 (questionnaire).

Multimedia Appendix 2 Quality of data records for Step 2 (physical measurement) and Step 3 (biochemical measurements).

Multimedia Appendix 3 Survey design weight.

Multimedia Appendix 4 Nonresponse weight adjustment for questionnaire and physical measurements.

Multimedia Appendix 5 Nonresponse weight adjustment for biochemical measurements.

Multimedia Appendix 6 Adjustment weights for age groups for each district for the questionnaire and physical measurements.

Multimedia Appendix 7 Adjustment weights for age groups for each district for biochemical measurements.

Multimedia Appendix 8 Adjustment weights for ethnicity for each study step.

## Abbreviations

EAs: enumeration areas
LiHep: lithium heparin
NaF: sodium fluoride
NCDs: noncommunicable diseases
PSU: primary sampling unit
STEPS: STEPwise approach to surveillance
VA: village area
WHO: World Health Organization
