# Supplementary material for: A National Surveillance Survey on Noncommunicable Disease Risk Factors: Suriname Health Study Protocol
Source: JMIR Res Protoc. 2015 Jun 17;4(2):e75. doi: 10.2196/resprot.4205 (PMC4526944; doi:10.2196/resprot.4205)
Supplement: Multimedia Appendix 1 [file resprot_v4i2e75_app1.pdf]

Personal lifestyle and risk factor information		Valid records	Records missing and not valid
**Demographic information**			
	Address	5748	0
	Sex	5748	0
	Age	5748	0
	Education	5652	96
	Ethnicity/Nationality/Religion	5719	29
	Relation	5721	27
	Working status	5698	50
	Income	4052	1696
Smoking		5724	24
Alcohol use		5709	39
**Nutrition**			
	Fiber consumption	5684	64
	Use of fats and oil	5589	159
	Use of sugary and fatty food	5705	43
	Dietary habits (N=1108)	1091	17
	Self-image	5701	47
	Salt use	5647	101
Physical activity		5689	59
**Economical issues**			
	Cost of health care	5628	120
	Productivity (N=1243)	1183	60
**Blood pressure**			
	History and practices	5709	39
**Diabetes**			
	History and practices	5691	57
**Mental health**			
	Kessler Psychological Distress Scale evaluation	5436	312
	Self assessment	5695	53
	Personal comfort	5682	66
**Health behavior**			
	Use of health care	5671	77
	Exposure to preventive measures and services	5660	88
