# Supplementary material for: A National Surveillance Survey on Noncommunicable Disease Risk Factors: Suriname Health Study Protocol
Source: JMIR Res Protoc. 2015 Jun 17;4(2):e75. doi: 10.2196/resprot.4205 (PMC4526944; doi:10.2196/resprot.4205)
Supplement: Multimedia Appendix 2 [file resprot_v4i2e75_app2.pdf]

Physical measurements (N=5748)		Records missing and not valid	Mean value	SD
	Length	5659	1.6197	0.0954
	Weight	5688	71.22	17.45
	Waist	5523	88.53	15.35
	Systolic blood pressure	5635	118.25	18.61
	Diastolic blood pressure	5645	78.44	12.23
	Heart beat	5606	79.08	11.99
**Biochemical measurements (N=3765)**				
	Low-density lipoprotein	3017	3.0682	0.93644
	Triglycerides	3022	1.2717	1.09458
	High-density lipoprotein cholesterol	3030	1.1825	0.31869
	Total cholesterol	3030	4.4968	1.04996
	Glucose	3323	5.9296	2.10018
