# Supplementary material for: A National Surveillance Survey on Noncommunicable Disease Risk Factors: Suriname Health Study Protocol
Source: JMIR Res Protoc. 2015 Jun 17;4(2):e75. doi: 10.2196/resprot.4205 (PMC4526944; doi:10.2196/resprot.4205)
Supplement: Multimedia Appendix 3 [file resprot_v4i2e75_app3.pdf]

District	Household design weight	Cluster design weight	Sample design weight
Paramaribo	23.4566	1.0707	25.1152
Wanica	29.6810	1.0000	29.6810
Nickerie	15.1185	1.1304	17.0904
Coronie	3.3569	1.8571	6.2338
Saramacca	8.8000	1.2222	10.7556
Commewijne	12.8369	1.0400	13.3504
Marowijne	4.9806	1.7500	8.7160
Para	6.7647	1.3077	8.8462
Brokopondo	7.1662	1.4444	10.3511
Sipaliwini	14.9766	1.0000	14.9766
