# Supplementary material for: A National Surveillance Survey on Noncommunicable Disease Risk Factors: Suriname Health Study Protocol
Source: JMIR Res Protoc. 2015 Jun 17;4(2):e75. doi: 10.2196/resprot.4205 (PMC4526944; doi:10.2196/resprot.4205)
Supplement: Multimedia Appendix 4 [file resprot_v4i2e75_app4.pdf]

Age group	Nickerie		Saramacca		Paramaribo		Commewijne		Marowijne	
	Men	Women	Men	Women	Men	Women	Men	Women	Men	Women
15-24	1.404	1.254	1.755	1.306	1.659	1.516	1.336	1.226	1.830	1.430
25-34	1.254	1.072	1.450	1.287	1.591	1.259	1.250	1.041	1.510	1.334
35-44	1.180	1.191	1.486	1.349	1.457	1.337	1.181	1.035	1.381	1.198
45-54	1.169	1.146	1.279	1.433	1.453	1.339	1.208	1.167	1.280	1.583
55-64	1.393	1.046	1.611	1.240	1.294	1.316	1.075	1.071	1.454	1.116
Age group	Coronie		Para		Wanica		Brokopondo		Sipaliwini	
	Men	Women	Men	Women	Men	Women	Men	Women	Men	Women
15-24	1.406	1.292	1.254	1.104	1.944	1.680	1.189	1.029	1.086	1.015
25-34	1.524	0.996	1.241	1.066	1.773	1.467	1.333	1.091	1.068	1.020
35-44	1.586	1.078	1.181	1.048	1.440	1.371	1.181	1.131	1.039	0.996
45-54	1.218	1.221	1.121	1.109	1.433	1.423	1.511	1.054	0.921	1.016
55-64	1.218	1.255	1.013	1.059	1.645	1.369	1.153	1.110	1.125	0.997
