# Supplementary material for: A National Surveillance Survey on Noncommunicable Disease Risk Factors: Suriname Health Study Protocol
Source: JMIR Res Protoc. 2015 Jun 17;4(2):e75. doi: 10.2196/resprot.4205 (PMC4526944; doi:10.2196/resprot.4205)
Supplement: Multimedia Appendix 5 [file resprot_v4i2e75_app5.pdf]

| Age group | Nickerie | | Saramacca | | Paramaribo | | Commewijne | | Marowijne | |
|---|---|---|---|---|---|---|---|---|---|---|
| | Men | Women | Men | Women | Men | Women | Men | Women | Men | Women |
| 15-24 | 1.872 | 1.803 | 3.259. | 2.067 | 2.982 | 2.542 | 1.902 | 1.839 | 2.563 | 2.013 |
| 25-34 | 2.100 | 1.481 | 3.383 | 1.716 | 2.961 | 1.955 | 4.687 | 1.945 | 2.454 | 1.769 |
| 35-44 | 1.475 | 1.315 | 2.686 | 2.231 | 2.980 | 2.355 | 2.450 | 1.655 | 2.436 | 1.424 |
| 45-54 | 1.347 | 1.371 | 1.692. | 2.149 | 2.552 | 2.108 | 2.472 | 1.677 | 2.133 | 2.500 |
| 55-64 | 1.811 | 1.157 | 2.187 | 1.635 | 2.324 | 1.989 | 2.315 | 1.707 | 2.237 | 1.396 |

| Age group | Coronie | | Para | | Wanica | | Brokopondo | | Sipaliwini | |
|---|---|---|---|---|---|---|---|---|---|---|
| | Men | Women | Men | Women | Men | Women | Men | Women | Men | Women |
| 15-24 | 2.460 | 1.723 | 2.759 | 2.023 | 2.592 | 2.204 | 3.447 | 1.759 | 2.456 | 1.467 |
| 25-34 | 2.287 | 1.280 | 2.628 | 1.644 | 2.604 | 2.109 | 3.998 | 1.795 | 2.957 | 1.294 |
| 35-44 | 1.903 | 1.317 | 2.303 | 1.592 | 1.885 | 1.880 | 2.717 | 1.388 | 1.700 | 1.114 |
| 45-54 | 1.740 | 1.487 | 1.827 | 1.613 | 2.425 | 2.160 | 3.625 | 1.265 | 1.094 | 1.115 |
| 55-64 | 1.623 | 1.434 | 1.893 | 1.569 | 2.684 | 1.966 | 1.467 | 1.295 | 1.243 | 1.069 |
