# Supplementary material for: A National Surveillance Survey on Noncommunicable Disease Risk Factors: Suriname Health Study Protocol
Source: JMIR Res Protoc. 2015 Jun 17;4(2):e75. doi: 10.2196/resprot.4205 (PMC4526944; doi:10.2196/resprot.4205)
Supplement: Multimedia Appendix 6 [file resprot_v4i2e75_app6.pdf]

Age group	Nickerie		Saramacca		Paramaribo		Commewijne		Marowijne	
	Men	Women	Men	Women	Men	Women	Men	Women	Men	Women
15-24	1.738	1.090	1.450	1.170	1.341	1.230	1.456	1.250	1.824	1.348
25-34	1.912	0.790	1.250	1.050	1.286	0.940	1.240	0.893	1.375	0.764
35-44	1.423	0.660	0.860	0.930	1.125	0.840	1.210	0.779	1.231	0.628
45-54	1.053	0.720	1.000	0.800	1.076	0.780	1.312	0.634	0.929	0.843
55-64	0.953	0.650	0.910	0.630	0.957	0.610	1.152	0.538	0.792	0.686
Age group	Coronie		Para		Wanica		Brokopondo		Sipaliwini	
	Men	Women	Men	Women	Men	Women	Men	Women	Men	Women
15-24	2.108	0.919	1.646	1.040	1.450	0.989	1.882	0.837	1.206	0.656
25-34	1.414	1.213	1.608	0.779	1.427	0.934	1.386	0.563	1.722	0.890
35-44	1.197	0.809	1.389	0.728	1.025	0.957	2.221	0.726	1.714	0.913
45-54	1.088	0.722	1.113	0.707	1.002	0.771	1.947	0.748	1.299	0.728
55-64	0.862	0.606	0.739	0.702	0.905	0.640	1.021	0.507	1.197	0.882
