# Supplementary material for: A National Surveillance Survey on Noncommunicable Disease Risk Factors: Suriname Health Study Protocol
Source: JMIR Res Protoc. 2015 Jun 17;4(2):e75. doi: 10.2196/resprot.4205 (PMC4526944; doi:10.2196/resprot.4205)
Supplement: Multimedia Appendix 7 [file resprot_v4i2e75_app7.pdf]

Age group	Nickerie		Saramacca		Paramaribo		Commewijne		Marowijne	
	Men	Women	Men	Women	Men	Women	Men	Women	Men	Women
15-24	1.783	1.121	1.447	1.174	1.340	1.231	1.456	1.249	1.839	1.359
25-34	1.522	0.740	1.246	1.046	1.286	0.935	1.240	0.893	1.386	0.770
35-44	1.459	0.681	0.859	0.925	1.125	0.841	1.210	0.779	1.240	0.633
45-54	1.080	0.740	1.004	0.804	1.076	0.783	1.312	0.634	0.936	0.778
55-64	0.978	0.680	0.906	0.630	0.957	0.606	1.152	0.538	0.798	0.691
Age group	Coronie		Para		Wanica		Brokopondo		Sipaliwini	
	Men	Women	Men	Women	Men	Women	Men	Women	Men	Women
15-24	2.108	0.919	1.647	1.039	1.441	1.007	1.920	0.854	1.206	0.656
25-34	1.414	1.213	1.609	0.779	1.419	0.928	1.418	0.574	1.721	0.890
35-44	1.197	0.809	1.389	0.727	1.033	0.951	2.265	0.740	1.714	0.913
45-54	1.088	0.722	1.113	0.707	0.996	0.775	1.346	0.764	1.299	0.728
55-64	0.862	0.606	0.739	0.702	0.900	0.635	1.042	0.518	1.197	0.883
