# Supplementary material for: A National Surveillance Survey on Noncommunicable Disease Risk Factors: Suriname Health Study Protocol
Source: JMIR Res Protoc. 2015 Jun 17;4(2):e75. doi: 10.2196/resprot.4205 (PMC4526944; doi:10.2196/resprot.4205)
Supplement: Multimedia Appendix 8 [file resprot_v4i2e75_app8.pdf]

**Ethnic group**	**Step 1 and 2**	**Step 3**
Creole	1.055664327	1.051831461
Hindustani	1.168565282	0.867602117
Javanese	0.84780732	0.922116494
Mixed	1.136233038	1.022042139
Maroon	0.884148122	1.102396507
Chinese	0.905062241	1.430637865
Kaukasian	0.905062241	1.430637865
Amerindian	0.905062241	1.430637865
No response for ethnicity	0.749869565	1.126151397
